# Acute Exercise and Academic Achievement in Middle School Students

**DOI:** 10.3390/ijerph16193527

**Published:** 2019-09-20

**Authors:** Andrew T. Harveson, James C. Hannon, Timothy A. Brusseau, Leslie Podlog, Charilaos Papadopoulos, Morgan S. Hall, EvaRose Celeste

**Affiliations:** 1 Department of Kinesiology/College of Health Science, California Baptist University, Riverside, CA 92503, USA; evarose.celeste@calbaptist.edu; 2 College of Education, Health, and Human Services, Kent State University, Kent, OH 44242, USA; jhannon5@kent.edu; 3 Department of Health, Kinesiology, & Recreation/College of Health, University of Utah, Salt Lake City, UT 84112, USA; tim.brusseau@utah.edu (T.A.B.); les.podlog@utah.edu (L.P.); morgan.hall@psych.utah.edu (M.S.H.); 4 Department of Kinesiology/College of Education and Kinesiology, Pacific Lutheran University, Tacoma, WA 98447, USA; papadoha@plu.edu

**Keywords:** physical activity, mathematics, Stroop test, exercise

## Abstract

(1) The purpose of this study was to examine the acute effects of aerobic exercise, resistance exercise, and non-exercise on measures of academic achievement and cognition in pre-adolescent students. (2) In a randomized crossover design, sixty-three participants with a mean age of 13.7 ± 0.47 years completed 20 min of aerobic exercise, resistance exercise, or non-exercise with a period of seven days between each bout. Immediately after each bout, participants were tested for academic achievement and cognitive performance. Academic achievement was assessed using standardized, age-appropriate mathematics tests. Cognition was measured using the Dot, Word, and Color tasks of the Stroop Test (Victoria version). (3) Participants scored significantly higher on the mathematics tests (F_1,62_ = 4.50, *p* = 0.038) and all elements of the Stroop Test (Dot: F_1,62_ = 8.14, *p* = 0.006; Word: F_1,62_ = 9.90, *p* = 0.003; Color: F_1,62_ = 7.57, *p* = 0.008) following acute resistance exercise as compared to non-exercise. Math test performance was not statistically different between the aerobic and resistance exercise treatments (F_1,62_ = 0.214, *p* = 0.645), but participants did perform significantly better on all elements of the Stroop Test following resistance exercise as compared to aerobic exercise (Dot: F_1,61_ = 25.82, *p* < 0.001; Word: F_1,62_ = 14.73, *p* < 0.001; Color: F_1,62_ = 20.14, *p* < 0.001). (4) Resistance exercise acutely influenced academic achievement and cognition in a positive manner. Such results add to the growing body of research that may support an increase in the prescription of varied exercise modalities within school settings for the purposes of improving academic performance and student health.

## 1. Introduction

Previous research has shown that students who participate in greater levels of physical activity (PA) show a trend towards increased academic achievement (AA), as measured by grades and cognitive test scores, compared to their less active peers [1]. Academic achievement has also been positively correlated with physical fitness, and increased involvement in sports and exercise [2,3]. Acute exercise has also shown modest, positive effects on a related construct known as executive function [4], a term used to broadly describe controlled cognitive outcomes and higher-level thought processing. In the existing literature, executive functions are frequently measured by constructs such as working memory, attention, planning, and speed of processing, cognitive processes inextricably involved in AA. It has been previously hypothesized that primary mechanisms of action underlying the ability of exercise to influence executive functions include increased cerebral blood flow and neurotransmitter release, neurogenesis, and upregulation of neurotrophic factors [4]. While the body of literature surrounding the impact of exercise on executive function continues to grow, the specific line of research focused on the influence of acute exercise on AA in youth retains a host of factors that must still be elucidated. One such question that must be addressed related to the direct influence of exercise on AA is in determining potential influence of varying exercise modalities. There have been several studies that have investigated the connections between aerobic exercise and increased AA in preadolescents and adolescents (grades 5–8). Typically, AA is examined through measurements of mathematical skills or reading ability [5], and the general trend seen in the existing literature indicates modest improvement in AA following aerobic exercise intervention. Such results are consistently demonstrated across sport and physical education (PE) [3], as well as participation in other forms of aerobic exercise such as walking and jogging [6].

While aerobic exercise has been shown to increase AA for adolescents, there is a paucity of similar research on the effects of resistance exercise on AA in youth participants. However, existing research does suggest that resistance exercise positively affects cognition in adolescents, adults, and seniors [7,8], as well as prospective memory in college-aged students [9]. Importantly, it has also been established that resistance exercise is safe for adolescent participants under supervision [10]. Together with the observed decline in youth PA [11], and the life-long health implications of brain development in children and adolescence [12], it is vital that research identifies additional interventions that could benefit the mental and physical health of these populations, concurrently. Thus, the purpose of this study was to compare the acute effects of aerobic exercise, resistance exercise, and non-exercise on measures of AA and cognition in eighth-grade boys and girls. The authors hypothesized that both aerobic and resistance exercise would have similar, positive effects on AA compared to the non-exercise control, as demonstrated through a standardized test of mathematics and a test of cognition. 

## 2. Materials and Methods 

Participants were eighth-grade students sampled from a middle school in the southwestern United States. Participants (*N* = 63, 57 boys, 6 girls, mean age 13.7 ± 0.5 years) were asked to provide written assent in conjunction with written consent from a parent or legal guardian. Participants were all apparently healthy, as defined by their enrollment in PE class, and able to participate in regular exercise. No other inclusion or exclusion criteria was applied. The study was conducted in accordance with American College of Sports Medicine (ACSM) ethical guidelines and following the rules of the Declaration of Helsinki. Institutional Review Board approval was granted by the University of Utah (IRB_00061661) and the Salt Lake City School District.

To test academic achievement, four 10-question math tests were conducted, with questions taken from the New York State Testing Program eighth-grade standardized exams [13]. Similar tests have been used in previous research [14] and the tests used in the present study had been shown to be reliable and valid, containing internal consistency coefficients of *r* = 0.85. Reliability data were not available for mathematics tests in the state where data collection took place. Thus, out-of-state exams were used. Eighth-grade mathematics teachers at the site of data collection were consulted to confirm that material on the proposed mathematics tests had been covered during the same school year. To prevent a practice effect, different questions were used for each test. Participants had to complete each 10-question test within five minutes to provide a realistic classroom setting. Cognition was measured using the Stroop Test (Victoria version). The Victoria version of the Stroop Test has been used to determine executive function and selective attention using three increasingly challenging tasks (Dot, Word, and Color tests) performed in quick succession [15] and is commonly used in research with participants ranging from children to adults. Participants were progressively required to name colored dots (Dot test), words printed in the same color as dots (Word test), and finally color words printed in non-corresponding colors (Color test). Each task contained 24 items and challenged participants to deal with an interference effect, which can slow reaction time.

Participants were required to perform one familiarization session on the mathematics test, Stroop test, and exercise protocols during regularly scheduled PE classes. After a period of seven days, the students were separated into three groups, with each group performing one session of aerobic exercise, resistance exercise, and non-exercise each over the span of three weeks in a randomized crossover design. Experiment conditions were varied to avoid an order effect (i.e., group 1 performed resistance exercise first, group 2 performed aerobic exercise first, group 3 performed the non-exercise control condition first), as illustrated in Table 1. Previous research has shown that the beneficial effects of exercise on executive function, as measured by event-related brain potential, peak within 40 min [16]. Thus, the tests of mathematics and cognition were administered between 5 and 20 min after each exercise session. The authors also felt that such a time-span following acute exercise would best mimic what would be seen in authentic classroom settings where exercise might be used to boost academic performance. The primary author and trained research assistants collected data.

Previous work by Alves et al. [7] laid the groundwork for the aerobic and resistance exercise protocols used in this study, which were designed to elicit moderate intensity activity levels. The resistance exercise protocol involved six exercises, and participants completed two sets of 15 repetitions in each of the following: squat, lunge, pushup, band pull down, band row, and overhead press. Initial loads were determined based on information gathered during the familiarization session. Weight was reduced or exercises were modified if participants were unable to complete 15 repetitions per set [17]. The complete resistance exercise session was 20 min in length, with one-minute rest breaks between sets. The aerobic exercise intervention was likewise composed of 20 min of walking or jogging around an indoor track at approximately 50–60% of the participants’ age-predicted heart rate maximum. The non-exercise control group consisted of participants viewing a sports-related DVD for 20 min. To ensure that participants remained inactive, seated, and did not fall asleep, students were monitored by the primary author, trained research assistants, and classroom teachers, in accordance with prior research protocols [17]. Exercise intensity was monitored immediately following each set of resistance exercise and at five-minute intervals during the aerobic exercise intervention, using Borg’s original Rating of Perceived Exertion (RPE) scale [18].

Repeated measures analysis of variance (ANOVA) was used to determine whether differences existed among the treatments. An alpha of 0.05 was used to determine statistical significance. All analyses were completed using SPSS 22.0 (IBM Corp., Armonk, NY, USA). 

## 3. Results

**Figure 1:**
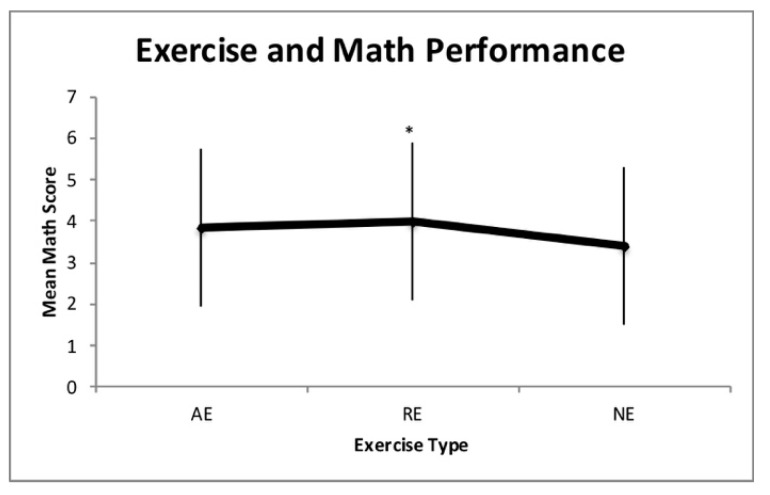
Mathematics performance following aerobic exercise (AE), resistance exercise (RE), and non-exercise (NE). * Denotes statistical significance (*p* < 0.05).

Analysis via repeated measures ANOVA showed significant differences in mean math test performance between resistance and non-exercise (F_1,62_ = 4.50, *p* = 0.038, η^2^ = 0.068). Differences in mean math test performance were statistically insignificant between aerobic exercise and non-exercise (F_1,62_ = 2.43, *p* = 0.124, η^2^ = 0.04), and aerobic exercise and resistance exercise (F_1,62_ = 0.214, *p* = 0.645, η^2^ = 0.003). See Figure 1 for the illustrated results.

**Figure 2:**
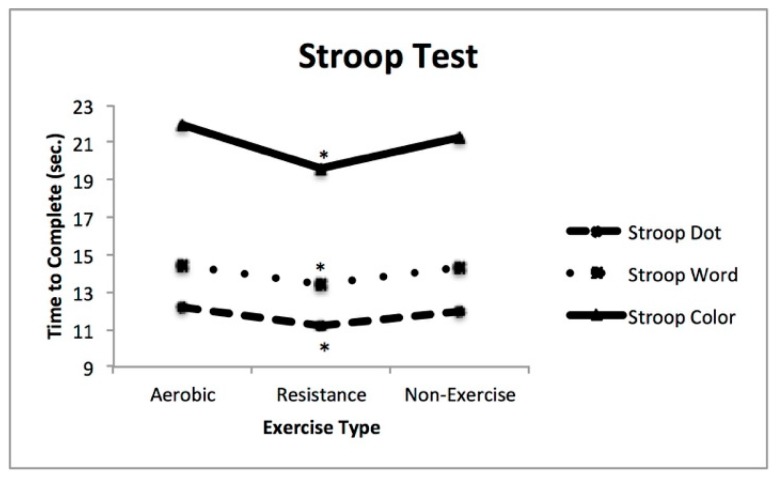
Time to complete Stroop Dot, Word, and Color tests following various exercise types. * Denotes statistical significance (*p* < 0.05).

Secondary analysis using repeated measures ANOVA revealed significant differences in mean scores between resistance exercise and non-exercise in the Stroop Dot test (F_1,62_ = 8.14, *p* = 0.006, η^2^ = 0.116), Stroop Word test (F_1,62_ = 9.90, *p* = 0.003, η^2^ = 0.138), and Stroop Color test (F_1,62_ = 7.57, *p* = 0.008, η^2^ = 0.109). Significant differences in mean scores were also found between resistance exercise and aerobic exercise in the Stroop Dot test (F_1,61_ = 25.82, *p* < 0.001, η^2^ = 0.294), Stroop Word test (F_1,62_ = 14.73, *p* < 0.001, η^2^ = 0.192), and Stroop Color test (F_1,62_ = 20.14, *p* < 0.001, η^2^ = 0.245). There were no significant differences between aerobic exercise and non-exercise across any of the Stroop Test elements. See Figure 2 for the illustrated results.

## 4. Discussion

The purpose of this study was to examine the acute effects of aerobic exercise, resistance exercise, and non-exercise on measures of AA and cognition in eighth-grade adolescents. The results supported the primary hypothesis that acute exercise has the potential to improve mean math test scores compared to non-exercise. Specifically, acute resistance exercise showed the greatest improvement in math scores as compared to aerobic and non-exercise conditions. To the authors’ knowledge, such findings are novel in that they mark the first time that acute resistance exercise has demonstrated a positive influence on AA in a middle school-aged sample. Aerobic exercise also improved mean math score by an average of 0.44 points (out of 10) and, while this result was not statistically significant, the authors believe an argument could be made that such an increase is indicative of practical significance (η^2^ = 0.04) [19]. 

Our secondary study purpose was to identify the acute effects of varying exercise types on cognition in eighth-grade students. Similar to what was seen with AA, cognitive performance as measured by the Stroop Dot, Word, and Color tests was significantly enhanced following resistance exercise as compared to aerobic exercise and non-exercise. Again, to the authors’ knowledge, such a result is a novel finding in a middle school-aged sample. To date, the cognitive benefits of acute resistance exercise have been demonstrated in high-school students [20], college students [9], as well as adult [8] and elderly subjects [21], indicating that there may be some unique mechanisms at play with the modality of resistance exercise and its ability to positively influence AA and cognition. It is the hope of the authors that such findings will provide added justification for the inclusion of resistance exercise as an exercise modality that can be used to improve middle school students’ academic performance and health simultaneously. 

While the exercise and its influence on the brain has been studied for quite some time, it is only recently that in-depth examinations of mechanisms involving acute exercise and its impact on AA and cognition have been explored. Despite the growth that is still occurring in this field of research, several hypotheses have been identified in previous literature that could offer insight into the findings illustrated in the present study. It is the authors’ belief that the neurotrophic stimulation hypothesis elucidated by Hillman et al. [22] is most applicable to our findings. This hypothesis states that neuromuscular activity stimulates areas of the brain that control executive function, resource allocation, and speed of processing. In light of the notable neuromuscular adaptations that take place following a regular resistance exercise routine, especially in its early stages [23], it is within reason to conclude that this hypothesis best explains why resistance exercise notably improved AA and cognitive performance over a more traditional aerobic exercise protocol and the non-exercise control. Further, acute resistance exercise has previously demonstrated an ability to significantly increase brain-derived neurotrophic factor (BDNF) [24], which is known to play a significant role in neuroplasticity [25]. Similarly, previous research indicates that the more complex nature of acute resistance exercise, as compared to more traditional aerobic exercise such as walking or jogging, may play a significant role in elevating cognitive response. Ozkaya et al. [21] found that cognition, as directly measured by event-related potentials (ERP) in the brain, was enhanced to a greater degree by resistance exercise as compared to both aerobic exercise and non-exercise. The authors postulated that the complexity of the resistance exercise tasks caused participants to employ greater attention to external stimuli, which up-regulated neurocognitive signaling. The sum of current evidence related to brain function supports the above theories and should not be disregarded as educators seek new ways to maximize student performance in the classroom. 

Finally, it would be remiss not to include the cerebral blood flow hypothesis as a potential mechanism that could potentially explain findings of the present study. The cerebral blood flow hypothesis states that during moderate exercise intensities of up to 60% maximal oxygen uptake (VO_2max_), the brain experiences an increase in blood flow and accompanying nutrients that positively enhance cognitive performance [26]. Such a mechanism should theoretically have enhanced AA and cognition similarly following both acute resistance and aerobic exercise in the present study, which were programmed at moderate intensities. It is also feasible that truly significant brain benefits are seen via synergism between increased cerebral blood flow and the neurotrophic factors identified previously. Additional research should look to isolate the precise mechanisms that drive improvements in executive function that are consistently seen throughout the current body of research surrounding acute exercise. 

Findings from the present study are certainly encouraging to those advocating the need for increased levels of physical activity in today’s schools. However, this study was not without its limitations, which must be noted. Likely, the most significant limitation was the absence of a precise measure of intensity during the exercise protocols. Student-reported RPE was collected during each bout of exercise and, while there were no statistical differences in RPE between protocols (F_1,61_ = 2.76, *p* = 0.102), it is possible that aerobic and resistance exercise were not precisely matched for intensity. Given the previously stated influence of cerebral blood flow on cognitive function, such a limitation may have skewed our results, and could explain the discrepant finding in the present study that aerobic exercise did not significantly improve cognition or AA, when it has consistently shown such benefits in past research. Further, while the non-exercise protocol was based on previous research, affect and arousal level were not directly measured, which could have potentially had a small influence on our results. An additional limitation of this study was the unequal representation of boys and girls. While it would have been desirable to have a representation that more closely matched the student body as a whole, the PE classes that were made available for sampling were comprised primarily of boys. Future studies should aim to more closely match the representation of sexes in order to minimize potential confounding variables.

## 5. Conclusions

In conclusion, the results of the present investigation indicate principally that acute resistance exercise can positively enhance AA in standardized math tests and cognition, as measured by the Stroop test, respectively. These findings are unique in that they are the first known, published examples of acute resistance exercise augmenting specific executive functions in an eighth-grade sample. Such findings should be used to encourage greater amounts and varieties of physical activity in the modern school system with hopes that the immediate and life-long benefits for student health and academic performance can best be realized.

## Figures and Tables

**Table 1:** Intervention order by group.

Intervention Group	Week 1	Week 2	Week 3
Group 1	Resistance	Aerobic	Non-Exercise
Group 2	Aerobic	Non-Exercise	Resistance
Group 3	Non-Exercise	Resistance	Aerobic
